# Preparation of Monoclonal Antibodies Against the gD Protein of Feline Herpesvirus Type-1 by mRNA Immunization

**DOI:** 10.3390/vetsci12070601

**Published:** 2025-06-20

**Authors:** Chengqi Zhang, Yawen Liu, Guangrong Zhao, Bo Hu, Liwen Xu, Jiajia Liu, Yajie Sun, Xiaolan Guo, Xiaoyu Deng, Shizhen Lian, Tiyun Han, Mengwei Xu, Shi Xu, Xue Bai

**Affiliations:** 1 Key Laboratory of Special Animal Epidemic Disease, Ministry of Agriculture, Institute of Special Animal and Plant Sciences, Chinese Academy of Agriculture Sciences, Changchun 130112, China; 2 Nanjing Chengshi BioTech (TheraRNA) Co., Ltd., Nanjing 210000, Chinaxumengwei@therarna.cn (M.X.)

**Keywords:** feline herpesvirus type-1, gD protein, monoclonal antibody, clinical diagnosis, mRNA vaccine

## Abstract

This study aimed to develop monoclonal antibodies (mAbs) against the gD protein of FHV-1 for rapid and specific virus detection. The gD protein, a highly conserved part of the FHV-1 envelope, is crucial for viral entry into host cells, making it an ideal detection target. We immunized BALB/c mice with an mRNA vaccine encoding the gD gene, achieving a serum antibody titer of 1:140,000 after three immunizations. The mice were then boosted with recombinant gD protein. Through cell fusion and multiple subcloning rounds, we obtained five hybridoma cell lines (D7, E4, E9, E10, and E19) that stably secrete anti-gD protein mAbs. Characterization by indirect immunofluorescence and Western blot showed that mAbs D7 and E4 have high specificity and strong binding activity against FHV-1, detectable at 2 μg/mL. These mAbs provide specific tools for FHV-1 detection and a basis for developing rapid diagnostic methods using ELISA, colloidal gold, and other technologies.

## Simple Summary

In this research, we designed and developed an mRNA vaccine targeting the feline herpesvirus type-1 (FHV-1) gD protein to immunize mice. Using hybridoma technology, we successfully generated monoclonal antibodies (mAbs) against the FHV-1 gD protein. The gD protein, essential for viral entry, was chosen due to its high conservation and immunogenicity. We obtained five hybridoma cell lines producing anti-FHV-1 gD protein mAbs, with D7 and E4 showing the highest specificity and binding activity. These antibodies serve as specific tools for FHV-1 detection and provide a basis for developing rapid diagnostic methods like ELISA and colloidal gold assays.

## 1. Introduction

Feline herpesvirus type-1 (FHV-1) is a key pathogen of feline upper respiratory disease (FURD) and poses an infection risk to cats of all ages. Particularly, kittens under six months of age have a high mortality rate after infection, which can exceed 50% [1,2]. Clinical symptoms of infected cats typically include fever, rhinitis (with serous or purulent discharge), conjunctivitis, and keratitis. When secondary bacterial infections occur, inflammation can be exacerbated, leading to severe complications, such as corneal perforation and, in extreme cases, panophthalmitis, resulting in permanent blindness. In recent years, it has been discovered that FHV-1 can also infect large felids, including Siberian tigers, South China tigers, cheetahs, and lions. The virus has now become a global threat to the health of both domestic cats and wild felines [3,4].

FHV-1 belongs to the subfamily Alphaherpesvirinae. It is a double-stranded DNA enveloped virus with a genome size of approximately 134 kb [5]. The virus structure consists of a lipid bilayer envelope that encloses 13 major glycoproteins [6]. Among these, gB, gD, gH, and gL are essential glycoproteins that play crucial roles in viral replication and infection, while gC, gE, gG, and gI are non-essential glycoproteins primarily involved in the replication process [7,8,9]. The gD protein, a key component of the viral envelope, is capable of recognizing and binding to receptors on host cells, thereby determining host cell tropism [10,11,12]. It also induces both humoral and cellular immune responses in the host. Due to its high conservation among glycoproteins and low variability, gD is considered the optimal antigen for FHV-1 detection [13].

FHV-1 is a major threat to felids and is widespread globally. FHV-1 infections are often complicated by secondary infections with pathogens like feline calicivirus (FCV), mycoplasmas, and chlamydiae. Traditional FHV-1 diagnostic methods, including virus isolation, electron microscopy, conventional PCR, and quantitative real-time PCR, are time consuming, complex, and demanding on operators, making them unsuitable for a rapid clinical diagnosis [6]. In recent years, mAbs have evolved from being research tools to being effective reagents in diagnostics and therapeutics, and are widely used in developing rapid detection methods such as the enzyme-linked immunosorbent assay (ELISA) and colloidal gold assays. Because mRNA vaccines trigger both humoral and cellular immunity, they are able to generate a more comprehensive immune response as well as cell-mediated neutralizing antibodies. Compared with inactivated vaccines, mRNA vaccines activate cell-mediated immunity more effectively, producing more cytotoxic T cells [14,15]. This study used an FHV-1 gD protein-producing mRNA vaccine to immunize mice. Through Western blot and indirect immunofluorescence, a high-sensitivity monoclonal antibody for FHV-1 detection was identified to aid diagnosis and research.

## 2. Materials and Methods

### 2.1. Cell Culture and Viral Infection

SPF-grade BALB/c mice were purchased from Changchun Yisi Laboratory Animal Technology Co., Ltd. (Changchun, China). The FHV, feline kidney fibroblast-like monolayer cell line (F81), ExpiCHO-S™ cells, and mouse myeloma cells (SP2/0) were all preserved by the Special Animal Disease Prevention and Control Innovation Team, Institute of Special Animal and Plant Sciences, Chinese Academy of Agricultural Sciences.

### 2.2. Design and Preparation of mRNA Vaccines

To ensure the successful expression of the gD protein, during the design of the mRNA sequence, the EPM and EABR sequences were inserted into the C-terminal end of the gD protein via a GS linker [16]. The pBluescript II KS(+) plasmid was employed as the in vitro transcription template for the gD gene (GenBank: YP_003331589.1). The plasmid was linearized by restriction enzyme digestion, and the linearized DNA template was purified using magnetic beads. The purity and integrity of the linearized DNA were confirmed by measuring the OD value and performing 1% agarose gel electrophoresis. The linearized plasmid was used as the template for in vitro transcription using a T7 in vitro transcription kit (TRANS, JT101-01). The cap analog, 7-methylguanosine, was incorporated directly into the transcription reaction mixture to ensure that the synthesized mRNA possessed a cap structure at the 5′ end. Following transcription, the residual DNA template was degraded using DNase I (ThermoFisher, 18047019, Waltham, MA, USA).

The synthesized mRNA was purified with an RNA purification kit (YEASEN, Gaithersburg, MD, USA, 12603ES96) per the manufacturer’s protocol to obtain naked mRNA for downstream use. Its concentration and purity were measured with a spectrophotometer, and its integrity was evaluated via capillary electrophoresis. Additionally, the vaccine’s polydispersity index and stability were assessed using a Zeta potential analyzer (Opptronix, Zhuhai, China). The FHV-gD protein mRNA vaccine was manufactured and provided by Nanjing Chengshi Biomedical Technology Co., Ltd. (Nanjing, China).

### 2.3. Preparation of mRNA–Lipid Nanoparticles

The preparation of mRNA–lipid nanoparticles (LNPs) was performed using microfluidic technology. The mRNA was mixed with four types of lipids: ionizable lipids, helper neutral lipids, cholesterol, and DMG-PEG2000. The molar ratio of these four lipids was 50:10:38.5:1.5, respectively, and the mass ratio of the mRNA to the total lipids (sum of the four lipids) was 1:6. The lipid mixture was dissolved in anhydrous ethanol, while the mRNA was dissolved in citrate buffer at pH 5.5. Rapid mixing was achieved using a microfluidic mixer. Through the precipitation of the lipids and electrostatic interactions, the mRNA was encapsulated within the LNPs to form the mRNA–LNP complex.

The optimized LNP formulation with a molar ratio of 50:10:38.5:1.5 was comprehensively characterized. The encapsulation efficiency was determined to be 94%, indicating a highly efficient loading of mRNA into the nanoparticles. The polydispersity index (PDI) of the LNPs was measured at 0.078, reflecting a narrow size distribution and uniformity of the nanoparticles. Furthermore, the storage stability assessments demonstrated that the LNPs remained stable for 6 months when stored at 2–8 °C, ensuring their prolonged functionality and usability.

### 2.4. Preparation of FHV Strains and Detection of Exogenous Viral Nucleic Acids

F81 cells were cultured in complete growth medium (MEM with 10% fetal bovine serum) until reaching 80% confluency. The culture medium was subsequently replaced with maintenance formulation (MEM containing 2% FBS), followed by viral inoculation with FHV-gD at a multiplicity of infection of 0.1. When 80% of the infected cells exhibited marked cytopathic effects, the culture supernatant was collected. Then, PCR technology was used to identify the pathogen and detect exogenous viruses in the samples to ensure no exogenous contamination of the viral solution. The primer sequences are shown in Table 1.

### 2.5. Detection of gD Protein Expression After mRNA Vaccine Transfection into HEK293T Cells

HEK293T cells were seeded in six-well plates at a density of 10^6^ cells per well and cultured overnight. When the cell confluence reached 80%, 2.5 µg of FHV-gD mRNA was transfected into the cells. The transfected cells were then incubated in a cell culture incubator at 37 °C with 5% CO_2_. After 24 h, FHV-gD protein expression was analyzed via Western blotting utilizing a 6xHis epitope-specific antibody (Servicebio, Wuhan, China, GB151251-100) [17].

### 2.6. Detection of Serum Antibody Levels and Neutralizing Antibody Titers in Mice After mRNA Vaccine Immunization

Female BALB/c mice (specific-pathogen-free, aged 6–8 weeks) were randomized into three experimental cohorts: mRNA vaccine (*n* = 3), licensed vaccine (*n* = 3), and PBS vehicle control (*n* = 3), with biological replicates maintained across groups. The mRNA group received 15 μg of gD gene mRNA vaccine per mouse; the commercial group, 100 μL of commercial vaccine; and the PBS group, 100 μL of PBS. Mice in the mRNA group were given two booster shots on Days 14 and 28 via subcutaneous neck injection. During the immunization period, mouse sera were collected every 14 days. Antibody titers were measured by indirect ELISA, and neutralizing antibody titers by in vitro assays.

Around 10^4^ F81 cells were seeded in each well of a 96-well plate and incubated at 37 °C in MEM with 10% FBS for 24 h. Mouse serum was serially diluted twofold in MEM and mixed equally with the FHV-1 virus (2 × 10^3^ TCID 50/mL). The mixture was incubated at 37 °C for 1 h and then inoculated into F81 cells at 100 μL per well. After 1 h, the medium was replaced with maintenance medium (MEM) containing 2% fetal bovine serum, and the cells were incubated for 3–4 days. The number of wells exhibiting CPE was counted for each serum dilution, and the serum neutralizing antibody titer was calculated using the Reed–Muench method.

### 2.7. Expression and Purification of gD Protein for Monoclonal Antibody Screening

The eukaryotic expression vector pcDNA3.4-gD encoding the FHV-1 gD protein was constructed using the pcDNA3.4 plasmid, with a 6xHis tag at the C-terminal end of the gD protein to aid recombinant protein purification. When the ExpiCHO-S™ cells reached a density of 4 × 10^6^ cells/mL (25 mL), 1920 μL of OptiPRO™ SFM was added to a 5 mL centrifuge tube. Then, 100 μg of the pcDNA3.4-gD plasmid was layered into the middle of the liquid and gently mixed by inversion. Next, 80 μL of ExpiFectamine™ CHO (ThermoFisher, Waltham, MA, USA) reagent was added to the center of the liquid and mixed again, followed by a 4 min incubation. The mixture was then added to the cell culture flask. After 24 h, a mixture of 150 μL of ExpiFectamine™ CHO enhancer and 6 mL of ExpiCHO™ (ThermoFisher, Waltham, MA, USA) supplement was added. Eight days post transfection, the supernatant was collected, and protein expression was detected using an anti-His tag antibody (Servicebio, Wuhan, China, GB151251-100) [17].

The gD recombinant protein was purified using Ni Sepharose 6 Fast Flow. The column was first flushed with five column volumes (CVs) of distilled water and then equilibrated with five CVs of equilibration buffer. Loading was performed at a flow rate of 1 mL/min. After loading, the column was washed with 20 CVs of wash buffer to remove impurities. Finally, the gD protein was eluted with five CVs of elution buffer, and the eluate was collected.

### 2.8. Flow Cytometry Detection of Mouse Lymphocyte Activation

On Day 28, two weeks after the second booster immunization, three mice were randomly selected from each group. Spleen cells were harvested to prepare cell suspensions, which were subsequently treated with red blood cell lysis buffer to isolate lymphocytes. The lymphocyte suspensions were then co-incubated with PE-conjugated CD4 (BioLegend, San Diego, CA, USA) antibody and APC-conjugated CD8 (BioLegend, San Diego, CA, USA) antibody at 4 °C for 30 min. After two washes, the cell samples were analyzed using a flow cytometer (FACS CytoFLEX S, Beckman, Shanghai, China). Data were analyzed using Flowjo V10 software.

### 2.9. Screening of Hybridoma Cells and Preparation of Ascites Antibodies

Fourteen days after the second immunization, three mice per group were randomly selected for flow cytometry of lymphocytes. After three rounds of mRNA vaccine immunization, mice with the highest serum neutralizing antibody titers were selected for intraperitoneal injection of 100 µg of recombinant gD protein to boost the immune response. Three days later, cell fusion was performed. Mice were euthanized under anesthesia, and splenocytes were harvested and fused with SP2/0 myeloma cells. The fused cells were cultured in 96-well plates using HAT medium, and cell growth was monitored. Once the cell confluence exceeded 80%, the culture supernatants were collected and screened for antibodies against gD protein using indirect ELISA. Hybridoma cell lines secreting antibodies specific to gD protein were identified and subjected to three rounds of subcloning to obtain stable monoclonal antibody-producing cell lines targeting the FHV-1 gD protein [18]. For ascites production, 10-week-old BALB/c mice were intraperitoneally injected with incomplete Freund’s adjuvant (0.5 mL per mouse). Three days later, the mice were inoculated intraperitoneally with hybridoma cells (1 × 10^6^ cells per mouse). When the mice exhibited significant abdominal swelling, ascites fluid was collected. The ascites fluid samples were centrifuged at 4000 r/min for 20 min, and the supernatant was purified using Protein G Sepharose 4 Fast Flow. The purified monoclonal antibodies were aliquoted and stored at −80 °C for further use.

### 2.10. Evaluation of the Binding Activity of Monoclonal Antibodies and Antibody Subtype Identification

FHV-1 was used to infect F81 cells, with uninfected cells as negative controls. When significant CPE emerged in the infected cells, the culture medium was abandoned. Primary antibodies from five hybridoma cell lines were applied and incubated at room temperature for an hour, and then the cells were washed thrice with PBST for 5 min each time. Next, goat anti-mouse IgG-FITC (Abcam, Cambridge, MA, USA, ab6785) as the secondary antibody was applied, followed by another hour of incubation and three 5 min washes with PBST. After washing, Mounting Medium, antifading with DAPI (Solarbio, Beijing, China, S2110) was added, and fluorescence was detected to evaluate the binding activity of five monoclonal antibodies targeting the conformational epitopes of FHV-1. Antibody binding to linear epitopes was assessed via Western blot using goat anti-mouse IgG-HRP (Abcam, ab205719). Monoclonal antibody subtypes were determined with a mouse antibody isotyping kit (Biodragon, Szczęsne, Poland, BF16002).

### 2.11. Virus Titration

FHV-1 titer was determined using the Reed–Muench method. Briefly, around 10^4^ F81 cells were seeded in each well of a 96-well plate and incubated at 37 °C in MEM with 10% FBS for 24 h. After washing, the cells were inoculated with a 10-fold diluted virus for 1 h. Then, the inoculum was removed, and the cells were maintained in MEM with 2% FBS for 3–4 days. CPE was monitored daily, and the TCID_50_ was calculated using the Reed–Muench method.

### 2.12. Statistical Analysis

Statistical analyses of the aforementioned data were performed using GraphPad Prism version 9 (GraphPad, San Diego, CA, USA). Statistical comparations were made by Student’s *t*-test, and *p*-values < 0.05 were considered significant (* *p* < 0.05,** *p* < 0.01, and *** *p* < 0.001).

## 3. Results

### 3.1. Characterization of the Physicochemical Properties of mRNA Vaccines

**Figure 1:**
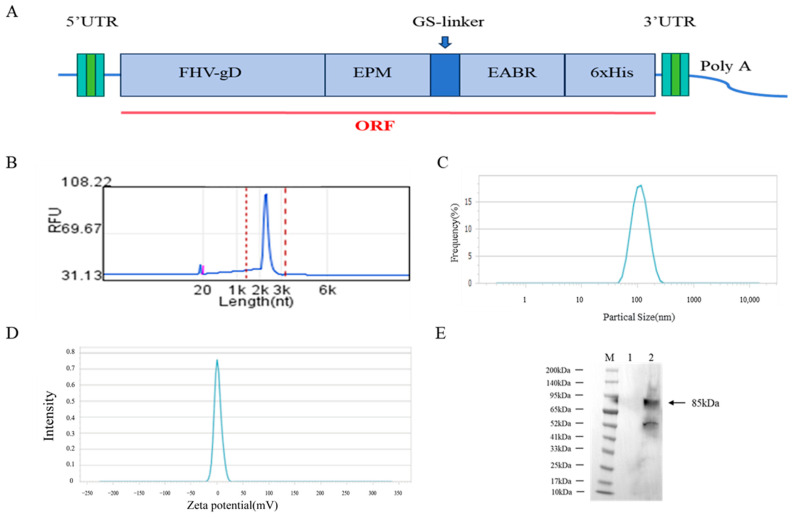
Design, preparation, and validation of protein expression of mRNA vaccine. (**A**) mRNA vaccine design schematic. (**B**) In vitro transcription mRNA quality and length determination. (**C**) Detection of the particle size and polydispersity index of mRNA–LNPs. (**D**) Zeta potential detection of mRNA–LNP vaccine. (**E**) Detection of gD protein expression in HEK293T cells transfected with mRNA–LNPs for 24 h. M represents the protein marker. Lane 1 contains untransfected HEK293T cells, and Lane 2 contains HEK293T cells transfected with mRNA–LNPs (original Figure see Supplementary Material).

The constructed mRNA sequence element was then cloned into the pBluescript II KS (+) vector containing the T7 promoter to generate an in vitro transcription (IVT) plasmid for mRNA production (Figure 1A). The mRNA was subsequently formulated into lipid nanoparticles (LNPs) to produce the mRNA–LNP vaccine. Capillary electrophoresis analysis revealed that the purified mRNA was of high purity and uniform size, with a sequence length of approximately 2000 nucleotides (Figure 1B). The mRNA–LNPs exhibited an average particle size of 110.96 nm (Figure 1C) and a polydispersity index (PDI) of 0.078, indicating uniform particle size distribution and a stable vaccine formulation.

### 3.2. Evaluation of mRNA Vaccine Immunization Effects and Expression of gD Protein for Monoclonal Antibody Screening

Proteins were extracted from HEK293T cells transfected with mRNA–LNPs, followed by protein purification. Western blot analysis was performed using an anti-6xHis-specific antibody. The results demonstrated that the FHV-gD protein was successfully expressed in HEK293T cells, with an apparent molecular mass of approximately 85 kDa (Figure 1E), consistent with the expected band.

### 3.3. Detection of gD Protein Expression for Monoclonal Antibody Screening

**Figure 2:**
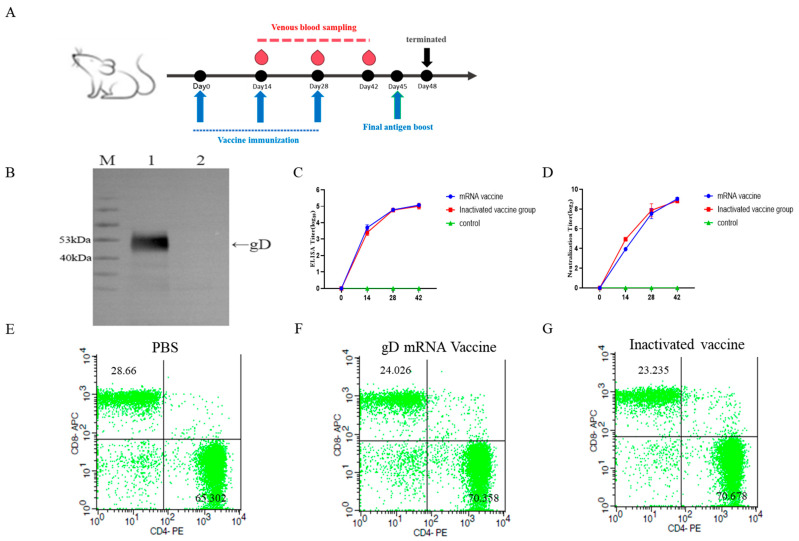
Evaluation of mRNA vaccine immunogenicity and antigen expression for monoclonal antibody screening. (**A**) Flowchart of mouse immunization procedure. (**B**) Expression of gD protein for monoclonal antibody screening. Lane 1: Supernatant from CHO cells transfected with pcDNA3.4-gD. Lane 2: Supernatant from CHO cells transfected with empty pcDNA3.4 vector. (**C**) Detection of FHV-specific antibody titers in mouse serum. (**D**) Detection of neutralizing antibody titers in mouse serum. (**E**–**G**) Flow cytometry analysis of splenocytes from immunized mice: Figure (**E**) represents the PBS control group, Figure (**F**) the mRNA vaccine group, and Figure (**G**) the inactivated vaccine group.

The plasmid pcDNA3.4-gD was transfected into ExpiCHO-S™ cells. The recombinant gD protein was purified using Ni Sepharose 6 Fast Flow, and Western blot analysis detected the expression of the target protein at approximately 50 kDa (Figure 2B).

### 3.4. Detection of gD Protein-Specific Antibody Levels and Neutralizing Antibody Titers in Mouse Serum

Figure 2A shows the immunization schedule and blood collection times for mice. Monoclonal antibody screening was performed using gD protein-coated ELISA plates to detect FHV-gD-specific antibody levels in mouse serum. Fourteen days after the first immunization, significant FHV-specific antibodies were detected in the mice. After three immunizations, the maximum ELISA titer reached 1:140,000 (Figure 2C), and the highest neutralizing antibody titer was 1:512 (Figure 2D).

### 3.5. Flow Cytometric Detection of Splenocyte Activation

Fourteen days after the secondary immunization, lymphocytes from mice in the mRNA vaccine, inactivated vaccine, and PBS control groups were analyzed using flow cytometry. Compared with the control group, both vaccine groups showed a significant increase in the proportion of CD4 + lymphocytes (*p* < 0.05) and a significant decrease in the proportion of CD8 + lymphocytes (*p* < 0.05) (Figure 2E–G).

### 3.6. Preparation and Screening of Hybridoma Cells

**Figure 3:**
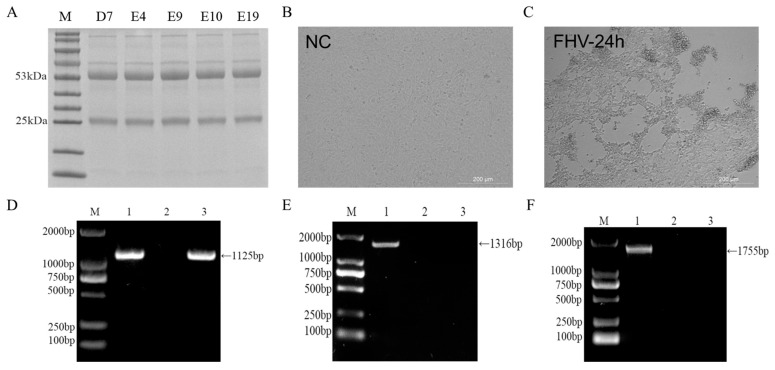
Monoclonal antibody purification and preparation of FHV. (**A**) SDS-PAGE analysis of five monoclonal antibodies. (**B**) Simulated FHV-1 infection of F81 cells and actual FHV-1 infection of F81 cells. (**C**) At 24 h post infection with FHV-1, F81 cells showed cytopathic effects, including cell shrinkage and detachment. (**D**) Nucleic acid testing for FHV in infected cell supernatant. Lane 1: Positive control. Lane 2: Negative control. Lane 3: Infected cell supernatant. (**E**) Nucleic acid detection of FCV in cell culture supernatant. Lane 1: Positive control. Lane 2: Negative control. Lane 3: Infected cell supernatant. (**F**) Nucleic acid detection in infected cell supernatant. Lane 1: FPV positive control. Lane 2: Negative control. Lane 3: Infected cell supernatant (original Figures see Supplementary Material).

After three immunizations, the serum ELISA titer of the mice reached 1:140,000, and the neutralizing titer reached 1:512 (Figure 2C,D), meeting the criteria for cell fusion. Three days after a booster immunization, splenocytes were harvested from the mice and fused with SP2/0 myeloma cells. Positive cell wells containing gD-specific antibodies in the supernatant were identified using indirect ELISA. Following three rounds of subcloning (Table 2), five hybridoma cell lines that stably secreted monoclonal antibodies against FHV-1 gD protein were obtained: D7, E4, E9, E10, and E19. These five cell lines were expanded for ascites production of monoclonal antibodies. The antibodies were purified using Protein G Sepharose 4 Fast Flow, and SDS-PAGE analysis revealed heavy chains at 50–55 kDa and light chains at 25 kDa (Figure 3A).

### 3.7. Preparation of FHV-1 Virus and Detection of Extraneous Viral Contamination

F81 cells were infected with FHV-1 at an MOI of 0.1. After 24 h (Figure 3C), significant cytopathic effects were observed, including cell shrinkage, rounding, and detachment. PCR tests on the cell culture supernatant were negative for FCV and FPV contamination (Figure 3D–F).

### 3.8. Characterization of Monoclonal Antibody Binding Properties by IFA

**Figure 4:**
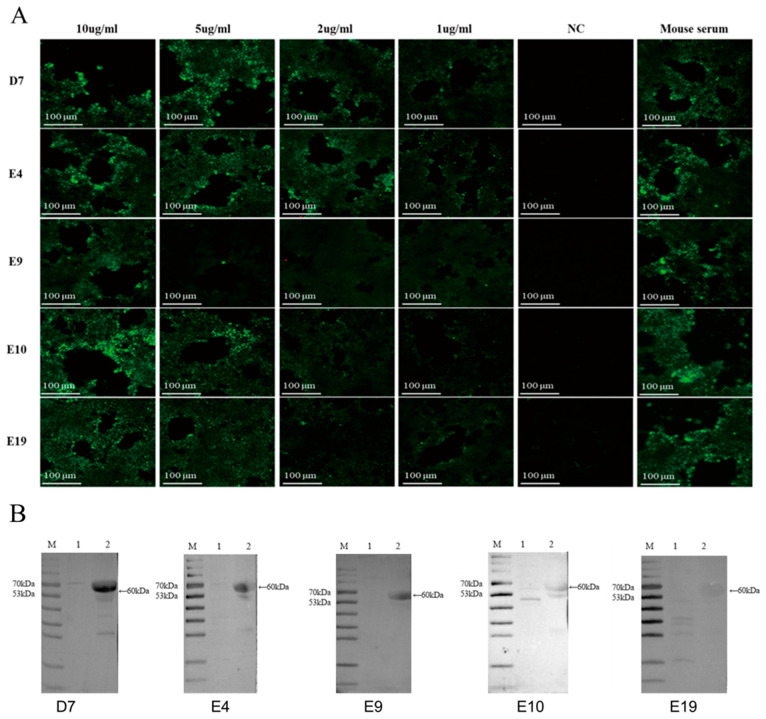
Monoclonal antibody binding activity assay. (**A**) Indirect immunofluorescence assay for detection of monoclonal antibody binding activity. (**B**) Western blotting assay for evaluating the binding activity of mAbs. M stands for protein Marker. Lane 1 is the F81 cell culture supernatant, and Lane 2 is the FHV viral solution (original Figures see Supplementary Material).

The FHV-1 strain was used to infect F81 cells, with uninfected cells serving as negative controls. After the infected cells exhibited significant CPE, the culture medium was discarded. Mouse ascites fluid antibodies from the five hybridoma cell lines were used as primary antibodies, and FITC-conjugated goat anti-mouse IgG (H+L) was used as the secondary antibody for an immunofluorescence assay (IFA). The results showed that infected cells exhibited distinct specific green fluorescence, while uninfected cells showed no fluorescence signal (Figure 4A). Among the five monoclonal antibodies, E4 and D7 exhibited higher titers, with visible fluorescence even at a dilution of 2 μg/mL.

### 3.9. Characterization of Monoclonal Antibody Binding Properties by Western Blotting

The FHV-1 strain was used to infect F81 cells, with uninfected cells serving as negative controls. After the infected cells exhibited significant CPE, the culture supernatant was collected. Western blot analysis was performed using five mAbs (2 µg/mL) as primary antibodies. The results demonstrated that mAbs D7, E4, E9, and E10 exhibited binding to the FHV-gD protein, as evidenced by specific bands at approximately 60 kDa (Figure 4B), consistent with the expected molecular weight of the target protein. Conversely, mAb E19 showed no detectable chemiluminescent signal in the Western blot assay. Among the five mAbs tested, mAb D7 demonstrated strong binding activity and high specificity in both IFA and Western blot analyses.

### 3.10. Monoclonal Antibody Subtype Analysis

Using the mouse monoclonal antibody subclass identification kit instruction manual, the subtypes of five monoclonal antibodies were determined. All five antibodies were of the IgM type and had kappa light chains. The results of the subtype identification and the heavy and light chain identification are shown in Table 3.

## 4. Discussion

FHV-1 is a major pathogen responsible for upper respiratory tract infections in felines. In clinical settings, it often co-infects with FCV and mycoplasmas, which not only exacerbates the clinical symptoms in infected cats but also increases the complexity of diagnosis [19,20]. Therefore, the development of effective and accurate diagnostic methods is crucial for the prevention and control of FHV-1 infections. Traditional diagnostic methods, such as virus isolation and PCR, are limited by their complex procedures and long turnaround times. In contrast, monoclonal antibody-based detection methods, including ELISA and colloidal gold immunochromatography, offer several advantages, such as high sensitivity, ease of operation, and high throughput. These methods are particularly suitable for rapid screening of large sample volumes and hold great potential for application in veterinary clinical diagnostics [21,22].

The FHV-gD protein is crucial for virus entry into host cells, especially during membrane fusion, viral particle release, and cell-to-cell spread [23]. As a key herpesvirus structural protein with a conservative amino acid sequence, gD is ideal for FHV antibody development [24,25,26]. The D7 monoclonal antibody, as selected in our experiments, shows good binding in the ELISA, Western blot, and indirect immunofluorescence assays. This indicates that it can bind well to both conformational and linear epitopes of the gD protein, making it suitable as a detection antibody for high-sensitivity test strips or kits.

FHV, as an enveloped virus, is released via budding after replicating in host cells. To enhance antigen display and boost immune response in mRNA vaccines, EPM and EABR sequences are added to the gD protein’s end. EPM, which prevents endocytosis, extends the protein’s cell surface presence, increasing antigen display and immune recognition. EABR recruits ESCRT proteins and thereby promotes virus-like particle formation and release, mimicking viral spread and enhancing intercellular antigen presentation and immune activation [27,28]. In flow cytometry assays, after two immunizations, the proportion of CD8^+^ T cells in mice from the mRNA vaccine group and the inactivated vaccine group decreased. This might be due to the differentiation of effector cells into memory cells after multiple immunizations, reducing the proportion of CD8^+^ T cells [29]. The decrease could also indicate transient immunological exhaustion from prolonged antigenic stimulation, which prevents excessive antigenic stimulation from harming the host [30]. CD4^+^ T cells, by differentiating into subsets, secreting cytokines, and regulating the functions of B cells and CD8^+^ T cells, maintain immune defense and homeostasis [31,32]. For instance, CD4^+^ T cells can promote B cell differentiation into long-lived memory cells via IL-6 and IL-10, establishing long-term immune protection. CD4^+^ T cells can also differentiate into Treg cells, which limit the overactivation of CD8^+^ T cells and NK cells through direct contact or inhibitory factor secretion, thus preventing immune-mediated damage [33]. In our experiment, both the commercial and mRNA vaccine groups showed an increased proportion of CD4^+^ T cells and a decreased proportion of CD8^+^ T cells. This might be due to immune tolerance from multiple immunizations in a short period. The rise in CD4^+^ T cells helps maintain immune balance and avoid tissue damage. Also, the increased CD4^+^ T cell proportion can boost B lymphocyte activation and conversion to long-lived memory B cells, which is beneficial for antibody secretion and the establishment of long-term immune protection.

IgM antibodies, known for their high affinity and multivalent binding, effectively target surface antigens. However, their short half-life and stringent storage conditions are drawbacks. In contrast, IgG antibodies offer greater stability. By fusing IgM and IgG structural domains via genetic engineering, the resulting hybrid antibodies combine IgM’s potent complement activation with IgG’s longer half-life, enhancing therapeutic efficacy [34,35,36]. Additionally, monoclonal antibodies with IgM–IgG fusion domains can be developed into diagnostic reagents with high sensitivity and specificity. Their robust antigen binding and stability improve antigen or biomarker detection accuracy in immune assays, aiding early disease diagnosis and clinical decision-making processes [37]. Monoclonal antibodies E4 and D7, being IgM antibodies, have shown good binding activity, high sensitivity, and specificity in indirect immunofluorescence and Western blot assays. They hold potential for developing fusion antibodies and offer promising prospects as more sensitive and accurate clinical detection tools.

In monoclonal antibody binding activity assays, indirect immunofluorescence and Western blot analyses were used to evaluate antibodies at various concentrations. Monoclonal antibody D7 was selected for its superior binding activity. It showed specific fluorescence even at a low concentration of <1 μg/mL and effectively bound to FHV-gD in Western blot at 2 μg/mL, making it a reliable tool for FHV clinical detection.

## 5. Conclusions

In this study, immunization with the FHV-gD-encoding mRNA vaccine successfully elicited FHV-specific antibodies and high-titer serum neutralizing antibodies in murine models. Through hybridoma technology, we generated five mAbs targeting FHV-gD protein, among which the D7 mAb demonstrated superior binding affinity. The D7 antibody exhibited strong reactivity in both indirect immunofluorescence assays and Western blot analyses, suggesting its potential utility as a diagnostic reagent for FHV detection. Notably, comparable neutralizing antibody titers were observed following immunization with both mRNA and inactivated vaccine platforms, indicating promising a prophylactic potential of mRNA-based formulations against FHV-1 infection. Further validation through immunization-challenge experiments will be required to confirm these preliminary findings.

## Figures and Tables

**Table 1:** Primer information.

Primer Name	Primer Sequence (5′→3′)	Amplified Size
FHV-1-F	ATGCATCATCATCATCATCATATGACACGTCTACATTTTT	1125 bp
FHV-1-R	TTAAGGATGGTGAGTTGTATGTATTATAGGAAGTTG	
FCV-F	CTCTGAGCTTCGTGCTTAAAACTCACA	1316 bp
FCV-R	GGGTTGTATGATTGCAT	
FPV-F	GAATTCGCCACCATGCATCATCATCATCATCATAGTGATGGAGCAGTTCAA	1755 bp
FPV-R	GAGCTCTTAATATAATTTTCTAGGTGCTAG	

**Table 2:** P/N values of hybridoma cell supernatants after three rounds of subcloning.

Hybridoma Cell Line		D7	E4	E9	E10	E19	Negative (OD_450nm_)
P/N	First subcloning	12.37818	5.418182	5.454545	3.841105	2.414669	0.1095
	Secondary subcloning	12.05802	14.5534	14.52913	10.35922	11.59021	0.1065
	Tertiary subcloning	32.25746	15.12088	20.53333	23.48571	36.83769	0.0965

**Table 3:** Identification of antibody subclasses.

	E4	E9	E10	E19	D7
Heavy chain	mu chain	mu chain	mu chain	mu chain	mu chain
Light chain	kappa	kappa	kappa	kappa	kappa
Antibody isotypes	IgM	IgM	IgM	IgM	IgM

## Data Availability

The data that support the findings of this study are available upon request from the corresponding author.

## Supplementary Materials

The following supporting information can be downloaded at: https://www.mdpi.com/article/10.3390/vetsci12070601/s1.
