# Supplementary figures and images for: Preparation of Monoclonal Antibodies Against the gD Protein of Feline Herpesvirus Type-1 by mRNA Immunization
Source: Vet Sci. 2025 Jun 20;12(7):601. doi: 10.3390/vetsci12070601 (PMC12298480; doi:10.3390/vetsci12070601)

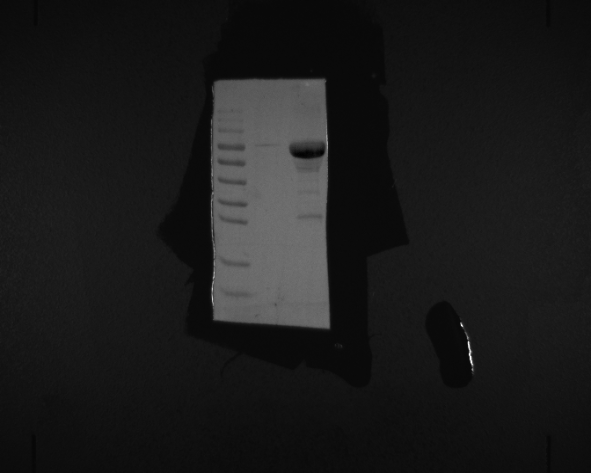

Supplement: Supplementary file 1 [file vetsci-12-00601-s001.zip › D7.tif]

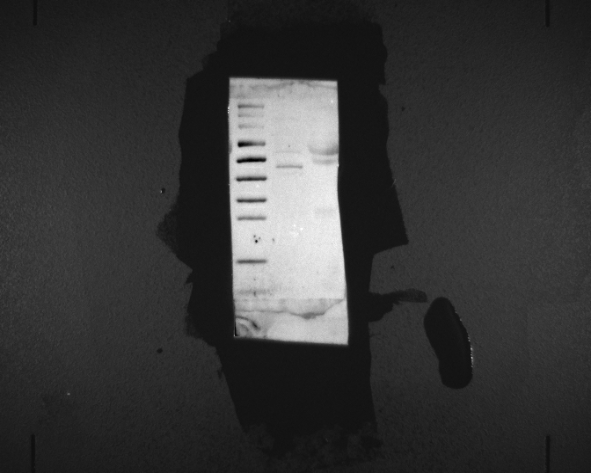

Supplement: Supplementary file 1 [file vetsci-12-00601-s001.zip › E10.tif]

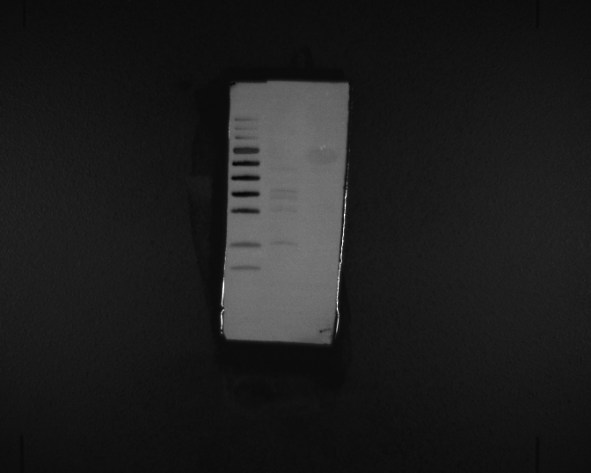

Supplement: Supplementary file 1 [file vetsci-12-00601-s001.zip › E19.tif]

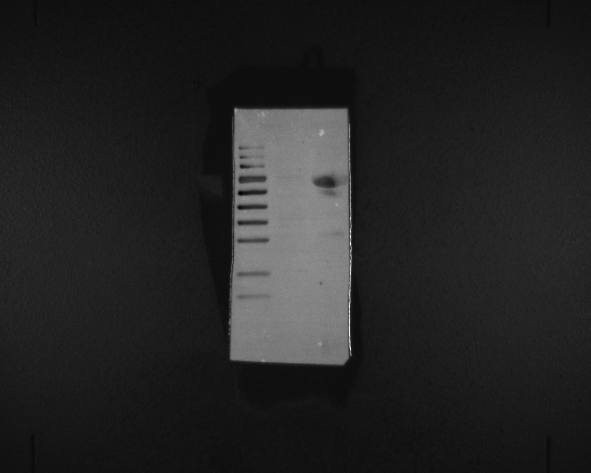

Supplement: Supplementary file 1 [file vetsci-12-00601-s001.zip › E4.tif]

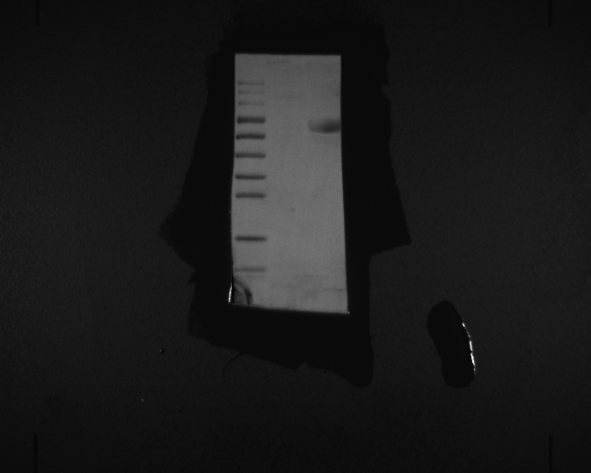

Supplement: Supplementary file 1 [file vetsci-12-00601-s001.zip › E9.tif]

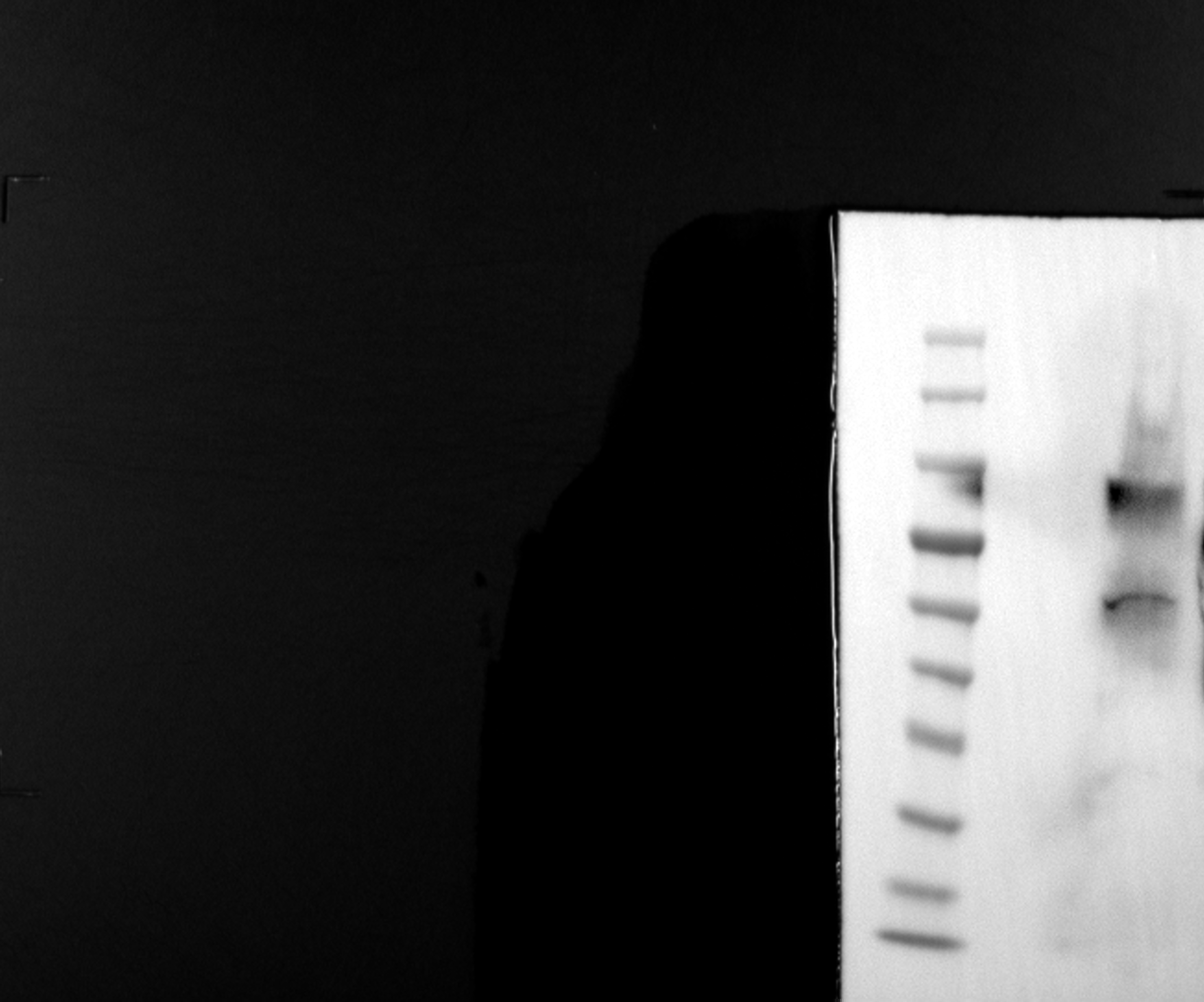

Supplement: Supplementary file 1 [file vetsci-12-00601-s001.zip › Fig1-E.tif]

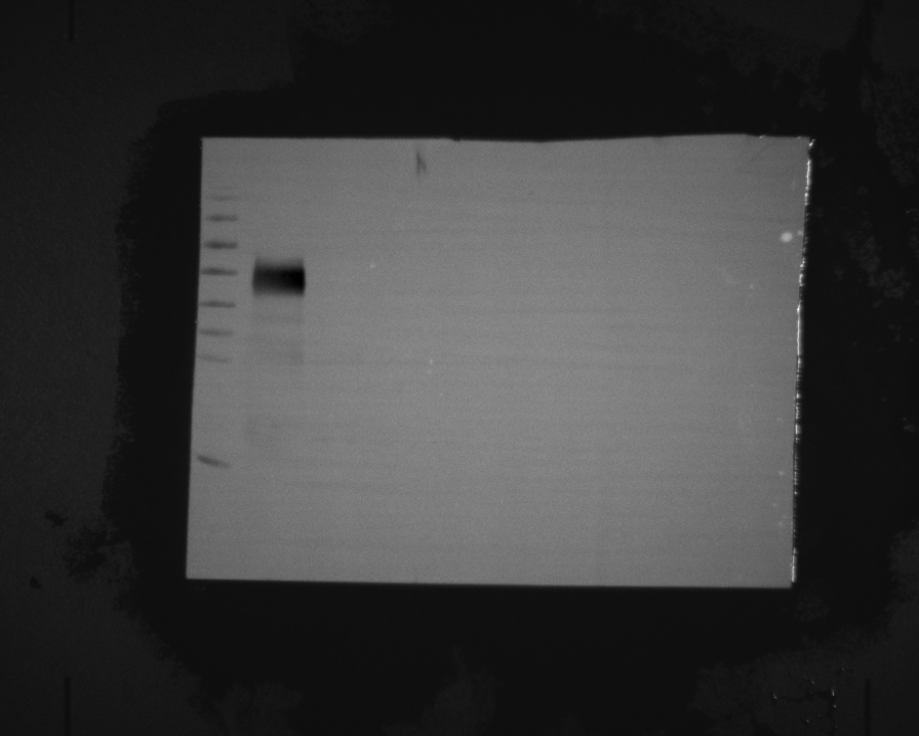

Supplement: Supplementary file 1 [file vetsci-12-00601-s001.zip › Fig2-B.tif]

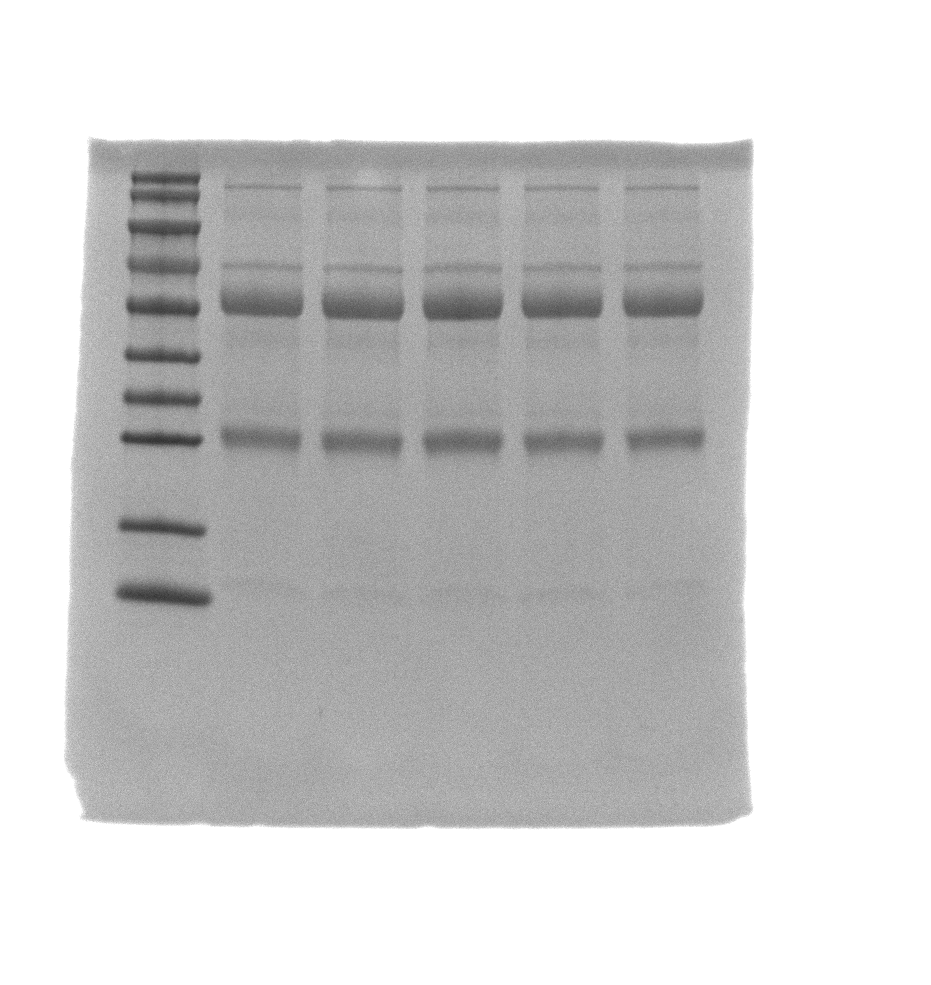

Supplement: Supplementary file 1 [file vetsci-12-00601-s001.zip › Fig3-A.tif]

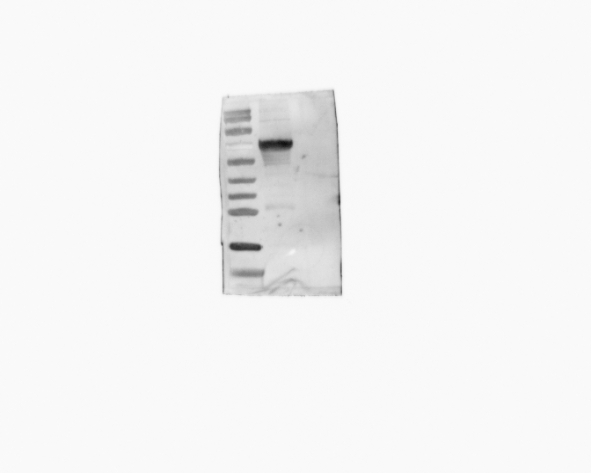

Supplement: Supplementary file 1 [file vetsci-12-00601-s001.zip › Fig4-B.jpg]

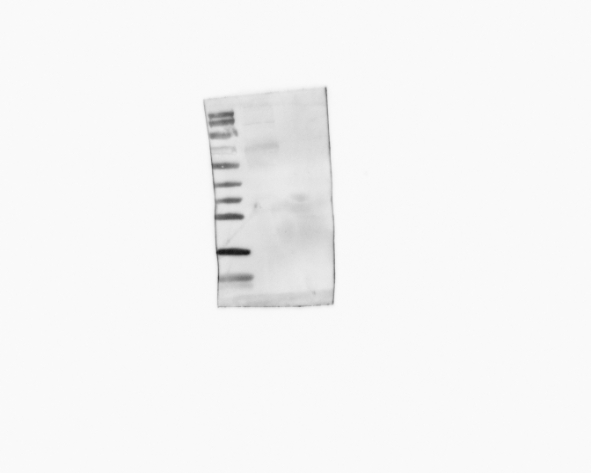

Supplement: Supplementary file 1 [file vetsci-12-00601-s001.zip › Fig4-C.jpg]

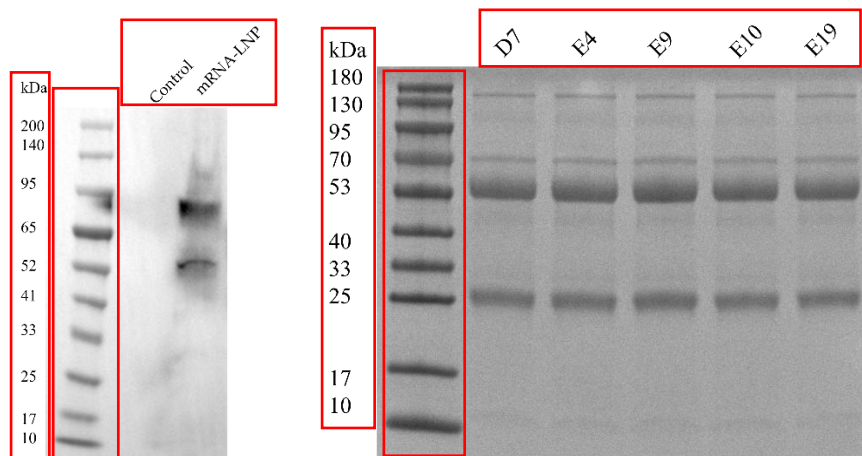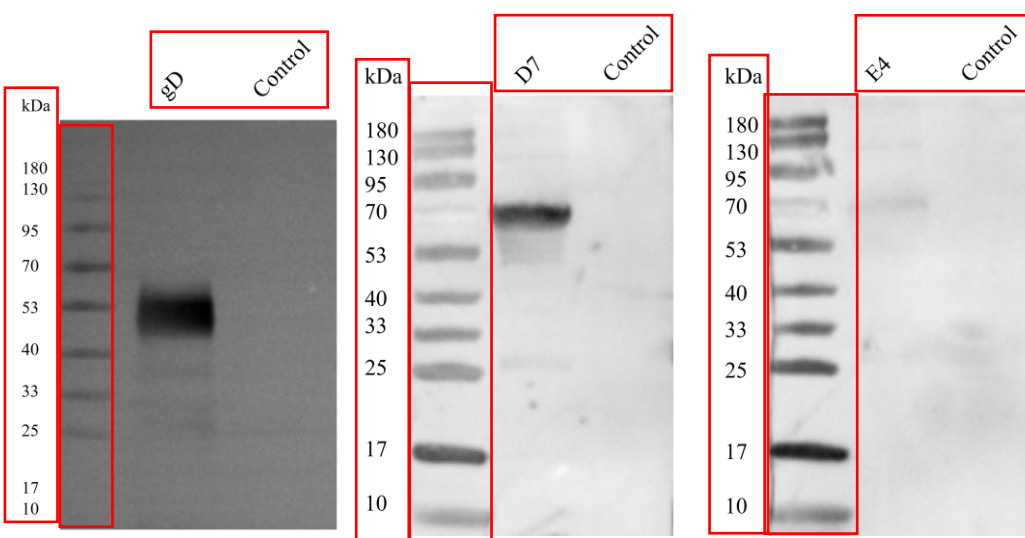

Supplement: Supplementary file 1 [file vetsci-12-00601-s001.zip › vetsci-3636509-supplementary.pdf]
